# Temporal and Spatial Regulation of CRE Recombinase Expression in Gonadotrophin-Releasing Hormone Neurones in the Mouse

**DOI:** 10.1111/j.1365-2826.2008.01746.x

**Published:** 2008-07

**Authors:** A Wolfe, S Divall, S P Singh, A A Nikrodhanond, A T Baria, W W Le, G E Hoffman, S Radovick

**Affiliations:** * Department of Pediatrics, Johns Hopkins School of MedicineBaltimore, MD, USA; † Department of Obstetrics and Gynecology, University of ChicagoChicago, IL, USA; ‡ Department of Pediatrics, University of ChicagoChicago, IL, USA; § Department of Anatomy and Neurobiology, University of MarylandBaltimore, MD, USA

**Keywords:** GnRH, LHRH, CRE recombinase, transgenic mouse

## Abstract

Gonadotrophin-releasing hormone (GnRH) neurones located within the brain are the final neuroendocrine output regulating the reproductive hormone axis. Their small number and scattered distribution in the hypothalamus make them particularly difficult to study *in vivo*. The Cre/loxP system is a valuable tool to delete genes in specific cells and tissues. We report the production of two mouse lines that express the CRE bacteriophage recombinase in a GnRH-specific manner. The first line, the GnRH-CRE mouse, contains a transgene in which CRE is under the control of the murine GnRH promoter and targets CRE expression specifically to GnRH neurones in the hypothalamus. The second line, the GnRH-CRETeR mouse, uses the same murine GnRH promoter to target CRE expression to GnRH neurones, but is modified to be constitutively repressed by a tetracycline repressor (TetR) expressed from a downstream tetracycline repressor gene engineered within the transgene. GnRH neurone-specific CRE expression can therefore be induced by treatment with doxycycline which relieves repression by TetR. These GnRH-CRE and GnRH-CRETeR mice can be used to study the function of genes expressed specifically in GnRH neurones. The GnRH-CRETeR mouse can be used to study genes that may have distinct roles in reproductive physiology during the various developmental stages.

Reproductive function is regulated by the activity of the hypothalamic–pituitary–gonadal axis. Gonadotrophin-releasing hormone (GnRH) is secreted by GnRH neurone axon terminals at the median eminence and regulates pituitary gonadotrophin synthesis and secretion. GnRH neurone cell bodies are primarily located in the basal forebrain with particular densities of neurones located in the diagonal band of Broca (DBB), the medial septal nuclei and the medial preoptic area (1–3).

Expression of the GnRH gene is anatomically very limited within the central nervous system. It is estimated that as few as 800 neurones express the gene in the mouse brain (4). Transgenic animal studies using various reporter genes have demonstrated that cell-specific expression of the mouse GnRH gene in the adult hypothalamus is regulated by the proximal 3446 base pairs of the mouse promoter (5–8).

Targeted gene disruption has been an important tool in elucidating the physiological role for a number of genes. However, this process produces animals in which all of the tissues and cells of the body possess the deleted gene. A number of investigators have deleted genes in specific tissue and cell types using the the Cre/*loxP* system. This system uses the targeted expression of the CRE bacteriophage recombinase to recognise consensus *loxP* target sequences that have been inserted into the gene of interest (9, 10). By crossing a mouse containing a transgene expressing CRE under the control of a cell-specific promoter with a mouse containing a gene flanked by *loxP* elements, an animal in which the desired gene is deleted only in the tissue of interest can be obtained. Site specific recombination using the Cre/LoxP system (11, 12) has been performed by a number of investigators to produce neurone (13), muscle (14), pancreatic β-cell (15, 16), kidney (17) and liver (16)-specific gene deletions in mice.

In the present study, we report the development of a transgenic mouse line that expresses the CRE bacterial recombinase in a GnRH neurone-specific manner. Using the 3446 bp mouse GnRH promoter fused to CRE cDNA, mice in which the CRE gene is expressed in GnRH neurones (GnRH-Cre) were produced. These mice are similar to mice produced in other studies (18–21). A transgenic mouse in which the transgene is engineered to be inducible by treatment with doxycycline allowing for temporal regulation of GnRH neurone specific Cre-mediated, conditional gene excision in mice (GnRH-CRETeR) was also developed. This allows for the gene recombination to occur at the desired time during the development of the mouse (22).

The production of mice containing GnRH neurone specific deletions of genes could prove valuable to our understanding of molecular and cellular elements that regulate GnRH neuronal development and function. The present study provides the initial characterisation of the two mouse lines.

## Materials and methods

### Transgene production

The GnRH-CRE transgene was produced by using the −3446/+28mGnRH promoter fragment (generous gift from Dr Donald DeFranco) cloned into pGEM11Z. A 1.7-kb fragment containing the CRE coding region, an intron derived from the SV40 *t-antigen* gene, and a nuclear localisation signal was extracted from the ACN cassette (23) by polymerase chain reaction (PCR) with an *Xho*I site added to the 5′ end and a *Hind*III site added to the 3′ end. The CRE gene was then inserted into the *Xho*I and *Hind*III sites of −3446/+28mGnRH-11Z. *Not*I and *Sal*I were used to linearise the transgene and to remove vector backbone.

The CRETeR vector was obtained from the laboratory of Dr Fred Wondisford (24). Briefly, the CRE fragment described above was amplified with *Hind*III ends by PCR and cloned into the *Hind*III restriction enzyme site within the multiple cloning site (MCS) of the pcDNA4/TO vector (Invitrogen, Carlsbad, CA, USA). The pcDNA4/TO vector contains a CMV promoter, with two Tet operators in the proximal promoter, 5′ of the MCS. On the 3′ side of the MCS is a bovine growth hormone polyadenylation sequence. A CMV-TetR cassette from pcDNA6/TO (Invitrogen) was then cloned downstream of CRE generating the CRETeR vector. The CRETeR vector was digested with *Bgl*II to release the CMV promoter, but retain part of the 2X Tet operator in the proximal promoter. The 3446 bp mouse GnRH promoter was amplified from the −3446/+28−11Z vector by PCR so that a *SgrA*I and a *BamH*I were added to the 5′ end. The 3′ end was designed to restore the 2X Tet operator by inserting into the *Bgl*II site with a *BamH*I end. This fragment was released from a shuttle vector by excision with *BamH*I and inserted into the CRETeR vector to produce GnRH-CRETeR. The vector was sequenced for verification and was linearised with *SgrA*I to remove vector backbone.

### Animal production

The linearised vectors were purified and injected into fertilised oocytes derived from a DC1 mouse to generate transgenic lines. Transgenic mice were identified by PCR and Southern blot analysis. Hybridisation probes were obtained by PCR using the primers (sense, 5′-ATGCCCAAGAAGAAGAGGAAGGTG-3′; antisense, 5′-TCGCGAGCTCGACCGAACAAA-3′) and the ACN cassette as a template producing a 1.3-kb fragment. Southern blot analysis was performed as previously described (25). PCR for genotyping was performed using the primers (sense, 5′-CGACCAAGTGACAGCAATGCT-3′; antisense, 5′-GGTGCTAACCAGCGTTTTCGT-3′). A 300-bp product will be produced in the presence of the transgene. Seven founder lines were produced for the GnRH-CRE transgene and four were produced for the GnRH-CRETeR transgene. Single founder lines were chosen based on immunohistochemistry and the remaining lines were sacrificed.

### Animal care and treatments

To induce CRE expression in the GnRH-CRETeR mice, doxycycline (DOX) was administered as a 5-mg pellet that was inserted dorsally with a 10-gauge trochar. The 21-day release doxycycline pellets were obtained from Innovative Research of America (Sarasota, FL, USA). For the examination of CRE expression, animals were sacrificed within 21 days to obtain tissue samples for histology. For the examination of CRE recombinase activity using the reporter mice, animals were sacrificed a minimum of 10 days after DOX capsule implantation. All experiments were approved by the Institutional Animal Care and Use Committees at Johns Hopkins Medical Institution, The University of Chicago or at The University of Maryland School of Medicine.

### R26R/GnRH-CRE or GnRH-CRETeR bitransgenic mice

To assess the anatomical distribution of functional CRE recombinase expression in the GnRH-CRE or GnRH-CRETeR mice they were crossed with the Gt(ROSA)26Sor^tm1Sor^ reporter mouse (R26R) developed by Soriano (26) and obtained from Jackson Laboratories (Bar Harbor, ME, USA). R26R mice were genotyped using three primers, p1, 5′-AAAGTCGCTCTGAGTTGTTAT-3′, p2, 5′-GCGAAGAGTTTGTCCTCAACC-3′, p3, 5′GGAGCGGGAGAAATGGATATG-3′. For the wild-type allele, primers p3 (sense) and p1 (antisense) will produce an approximately 550-bp amplicon. For the R26R allele, primers p2 (sense) and p1 (antisense) produce an approximately 300-bp amplicon. GnRH-CRE-TeR mice were also crossed with a related reporter mouse, the Gt(ROSA)26Sor^tm1(EYFP)Cos^ mouse (27) (a generous gift of Frank Costantino, Columbia University) to confirm the results in two separate reporter lines. Mice were sacrificed at between 3 and 5 months of age for histological analysis.

### Western blot analysis

GnRH-CRE mice were euthanised and hypothalamus, cerebral cortex, cerebellum, liver, heart and lung were dissected and place into RIPA buffer (50 mm Tris-HCl (pH 7.4), 150 mm NaCl, 1% NP40, 1 mm ethylenediaminetetraacetic acid, 1 mm phenyl-methylsulfonyl fluoride, 1 mm Na_3_VO_4_, 1 mm NaF, 40 μl/ml Protease Inhibitor Cocktail (Roche Molecular Biochemicals, Indianapolis, IN, USA) for western blot analysis. Tissues were homogenised with a Polytron tissue homogeniser (Brinkmann Instruments Inc., Westbury, NY, USA).

For hypothalamic dissections, a single fragment was taken consisting of tissue from 1 mm caudal to the mammillary bodies, rostrally to the olfactory bulbs, 1 mm laterally beyond the lateral aspect of the median eminence, and 3 mm dorsally.

Extracts were size fractionated by SDS-PAGE on an 8% resolving gel and transferred to Protran (Perkin Elmer, Waltham, MA, USA) nitrocellulose membrane by semi-dry transfer. Blots were stained with Ponceau solution [0.1% (w/v) Ponceau S in 5% acetic acid] to visualise protein transfer. Polyclonal antibody specific for CRE (Covance Research Products, Denver, PA, USA) was used. Blots were then incubated with HRP conjugated secondary antibodies (secondary antibodies obtained from Jackson Immunoresearch, West Grove, PA, USA). Antibody binding was visualised using the ECL Plus reagent (Amersham Pharmacia, Piscataway, NJ, USA).

### Histology

All animals were anaesthetised with 100 mg/kg pentobarbital ip, administered 10 IU heparin and perfused transcardially with saline containing 2% sodium nitrite followed by 2.5% acrolein in buffered 4% paraformaldehyde (28). After fixation, all brains were sunk in 25% aqueous sucrose and sectioned on a freezing microtome into 25 μm sections that are saved in 12 wells so that each well contains every twelth section. Sections were stored in cryoprotectant antifreeze (29) until staining was initiated.

Sections were removed from the cryoprotectant antifreeze, rinsed in potassium phophate-buffered saline (KPBS, 0.05 m phosphates, 0.9% NaCl, pH 7.4), treated with a 1% NaBH4 solution (Sigma, St Louis, MO, USA), rinsed, and then sections were incubated with anti-β-galactosidase antibody (rabbit polyclonal, Chemicon Temecula, CA, USA; dilution 1 : 500 000), anti CRE recombinase (Rabbit Polyclonal, Covance, Berkley, CA; USA; dilution 1 : 2 000 000), or anti-GnRH (rabbit polyclonal LR-1 gift of Dr Roger Guillemin and Dr Robert Benoit; dilution 1 : 150 000) in KPBS with 0.4% Triton X-100, for 48 h at 4 °C. After rinsing, the tissue was incubated for 1 h at room temperature in biotinylated horse anti-rabbit IgG (heavy and light chains; Vector Laboratories, Burlingame, CA, USA) at a concentration of 1 : 600 in KPBS with 0.4% Triton X-100, rinsed, and incubated for 1 h in avidin-biotin complex solution (‘elite’ ABC kit, Vector Laboratories; 4.5 μl each per ml incubation mixture). Visualisation was performed using DAB either alone or with nickel salts (28).

Double-labelling was performed as previously reported (30–32). Briefly, the CRE staining was performed first. After rinsing in KPBS then in 0.175 m sodium acetate (NaOAc), the antibody-peroxidase complex was visualised with a solution of NiSO4 (25 mg/ml), 3,3-diaminobenzidine HCl (NiDAB, 0.2 mg/ml), and H_2_O_2_ (0.83 μl of a 3% solution per ml of final mixture) in aqueous 0.175 m NaOAc that yielded a blue-black reaction product that was confined to the nucleus. In some cases, sections were treated with a β-Gal Staining Set (Roche) to assess enzymatic activity. Sections were then stained for GnRH as described for the single label above. The tissue was rinsed and reacted using Tris saline (0.1 m solution pH 7.2) and DAB without nickel salts as the chromogen (28). This procedure provided a brown reaction product. After approximately 15–20 min, the tissue was rinsed in the acetate solution or Tris-saline solution, rinsed in KPBS, and then placed into saline. The sections were mounted onto subbed slides, air-dried overnight, dehydrated in alcohols, cleared in xylenes and mounted with Histomount and coverslipped. Cell counts were performed on sections ranging from the caudal olfactory bulb to the caudal hypothalamic aspect.

## Results

### GnRH-CRE and GnRH-CRETeR mice are fertile

**Fig. 1:**
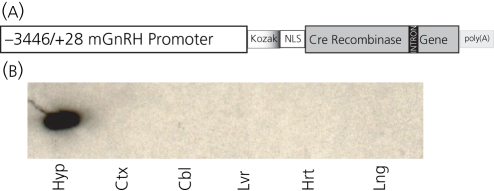
Targeting of CRE recombinase to the hypothalamus of transgenic mice. (a) The construct used to target CRE recombinase is illustrated. A −3446 bp fragment of the mouse gonadotrophin-releasing hormone (GnRH) promoter was fused upstream of CRE recombinase. CRE recombinase gene contains a Kozak initiation site, a nuclear localisation signal, an intron, derived from the SV40 *t-antigen* gene and a poly(A) signal. (b) Western blot analysis using an antibody specific for CRE protein in the indicated tissues from a GnRH-CRE mouse, founder line 35. Tissues shown are hypothalamus (hyp), cerebral cortex (ctx), cerebellum (cbl), liver (lvr), heart (hrt) and lung (lng).

**Fig.4:**
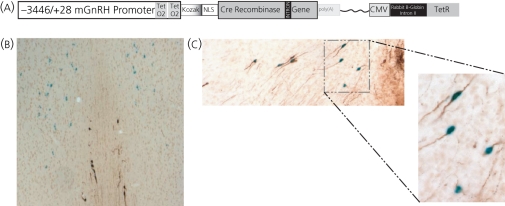
Gonadotrophin-releasing hormone (GnRH) neurones exhibit CRE recombinase activity in doxycycline treated GnRH-CRETeR mice. (a) The construct used to produce the GnRH-CRETeR mouse is shown. A −3446 bp fragment of the mouse GnRH promoter was fused upstream of CRE recombinase as for the GnRH-CRE mouse. The proximal region of the GnRH promoter was modified to insert two Tet operators (TetO2). In series, the transgene also contains the CMV promoter regulating the Tet repressor gene. The rabbit β-globulin intron II is inserted between the promoter and the gene to enhance expression of the TetR gene. (b) Coronal sections from a bitransgenic GnRH-CRETeR/ROSALacZ mouse treated with Dox. Section includes the medial and lateral septum and the DBB. Tissue was treated with X-gal to visualise β-galactosidase activity and is seen as blue. GnRH neurones were stained as above and are brown. A population of X-gal stained neurones are seen in the lateral septum and do not express GnRH. Nine GnRH expressing neurones are seen in the medial septum and DBB, eight of which colocalised with X-gal staining. (c) GnRH neurones at the level of the organum vasculosum of the lamina terminalis/preoptic area at × 100 magnification. Ten GnRH immunostained neurones are depicted and nine co-stain blue indicating CRE recombinase activity. The boxed region is further displayed at × 250 magnification.

Seven founders were produced that contained the GnRH-CRE transgene (Fig. 1a) and four founders were produced that contained the GnRH-CRETeR transgene (Fig. 4a). Two of the GnRH-CRE founder mice did not transmit the transgene to their offspring and were sacrificed. All of the remaining GnRH-CRE and GnRH-CRETeR founder mice transmitted their transgene to their offspring. Mice from each of these lines were bred for several generations and exhibited normal fertility compared to other mice in our colony, indicating that the expression of CRE in GnRH neurones did not impair reproductive function for the GnRH-CRE mouse line. For this reason, it is expected that CRE will not impact fertility in the Dox treated GnRH-CRETeR mice, although this has yet to be tested.

### CRE protein is localised to the hypothalamus in GnRH-CRE mice

To determine the specificity of CRE expression, a western blot analysis was performed using homogenised tissues in GnRH-CRE mice from the hypothalamus, cortex, cerebellum, liver, heart and lung. Fig. 1(b) shows that CRE protein was only observed in the hypothalamic tissue sample where GnRH neurones are localised.

### CRE protein is localised to GnRH neurones in GnRH-CRE mice

**Fig. 2:**
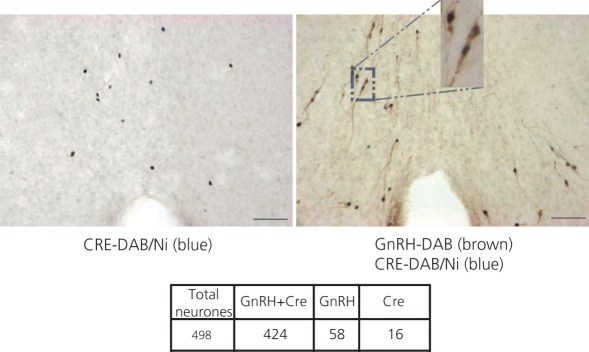
CRE recombinase is expressed in gonadotrophin-releasing hormone (GnRH) neurones. Coronal section of a brain from a single 4-month-old male GnRH-CRE mouse (founder 35). Both sections are hypothalamic immediately caudal to the level of the organum vasculosum of the lamina terminalis. On the left is staining for Cre alone with DAB/Ni showing nuclear blue staining. On the right is an adjacent section stained for Cre (blue nuclear) and for GnRH (brown cytoplasmic). Both of these images are at × 100 magnification. Several neurones are boxed and shown in the inset at × 250 magnification. Scale bars = 100 μm. Cell counts from all brain sections from this mouse are also shown.

To specifically localise CRE protein to GnRH neurones, double labelling immunohistochemistry was performed on coronal sections of mouse brains obtained from GnRH-CRE mice. A representative section from a 4-month-old male mouse (no. 6102) is shown in Fig. 2. On the left of Fig. 2, immunostaining is shown using only the polyclonal CRE antibody. Nuclear CRE staining (blue) is observed in cells located in the olfactory tissues, ventral diagonal band of Broca, medial preoptic area, and at the level of the organum vasculosum of the lamina terminalis, all regions where GnRH neurones are located (1, 4). On the right of Fig. 2, double-labelling with the polyclonal CRE antibody and the LR1 polyclonal GnRH antibody is shown. CRE again was observed as blue nuclear staining. GnRH appears as brown cytoplasmic staining. An inset shows three of the double-labelled neurones at × 250 magnification. Cell counts were performed and 85% of stained neurones expressed both CRE and GnRH (424 out of 498). 11.6% (n = 58) of GnRH stained neurones did not clearly express Cre, and 3.2% (n = 16) of CRE stained neurones did not express GnRH (Fig. 2). Brains from three other GnRH-CRE mice from this same founder line (two female, no. 9721 and no. 8355 and one male, no. 357) were analysed as well and counts performed on every six sections (as are all subsequent analyses) indicated that similar percentages of GnRH neurones were colocalised with CRE (no. 9721, 64 of 82; no. 8355, 60 of 69; and no. 8357, 71 of 88) and that, similarly, few CRE expressing neurones were identified that were not GnRH neurones (no. 9721, four neurones; no. 8355, two neurones; no. 8357 and no. 8357, two neurones). Brains obtained from other founders were found to have equal or lesser degrees of colocalisation. These founders were ultimately sacrificed.

### Cre-dependent recombination is observed in GnRH neurones

**Fig. 3:**
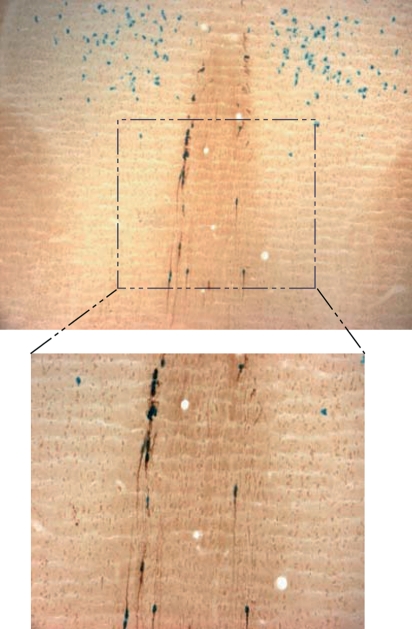
Gonadotrophin-releasing hormone (GnRH) neurones exhibit CRE recombinase activity. Coronal sections (× 100 magnification) from a bitransgenic GnRH-CRE/ROSALacZ mouse. Section includes the medial and lateral septum and the diagonal band of Broca (DBB). Tissue was treated with X-gal to visualise β-galactosidase activity, seen as blue. GnRH neurones were stained as above and are brown. A population of X-gal stained neurones are seen in the lateral septum and do not express GnRH. A group of GnRH expressing neurones are seen in the medial septum and DBB, all of which colocalise with X-gal staining. The boxed region is further shown at × 250 magnification.

To determine the extent to which CRE expression resulted in CRE recombinase activity, GnRH-CRE mice were crossed with the ROSA26 reporter mouse in which a loxP flanked stop cassette prevents constitutive expression of the β-galactosidase gene. Excision of the stop cassette results in β-galactosidase gene expression in a cell-specific manner (26). Histological staining for LacZ in a cell indicates that CRE recombinase activity has occurred. Histological analysis of six bitransgenic mice (no. 748 male, no. 752 male, no. 753 male, no. 751 female, no. 754 female and no. 755 female) aged 3–5 months indicated that 87% (416 of 476 neurones; 79.3 ± 5.65 GnRH neurones per mouse and 69.3 ± 5.14 neurones double-labelled per mouse) of GnRH neurones expressed LacZ, and thus CRE activity at some period during development (Fig. 3). This percentage was similar to the percentage of GnRH neurones expressing CRE in the adults examined (85%) (Fig. 2), suggesting that CRE expressing GnRH neurones had exhibited Cre-recombinase activity. There was also a population of LacZ expressing cells observed in the lateral septum that did not express GnRH (Fig. 3). These cells have also been observed by other investigators in GnRH-neurone specific CRE mice (19, 21) as well as those using other reporter systems (6, 33).

### CRE recombinase activity is localised to GnRH neurones in doxycycline treated GnRH-CRETeR mice

**Fig. 5:**
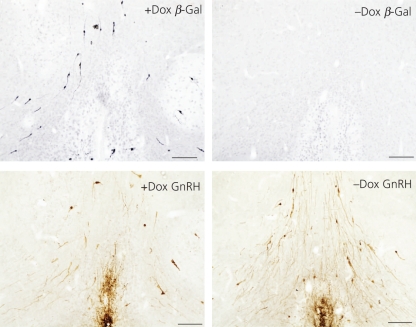
CRE recombinase activity is induced by doxycycline in the gonadotrophin-releasing hormone (GnRH)-CRETeR mouse. Coronal sections from a bitransgenic GnRH-CRETeR/ROSALacZ mouse treated with Dox (left, +DOX) and untreated (right, -DOX). The upper sections are stained for β-galactosidase (blue/black) and the lower sections have been stained for GnRH (brown). Scale bars = 100 μm.

GnRH-CRETeR mice were crossed with the ROSA26 or ROSA26YFP reporter mice. Bitransgenic mice aged 2–4 months were treated with a doxyxyline capsule or left untreated. Mice were sacrificed a minimum of 10 days following the insertion of the capsule. Because recombinase activity, once complete, is not reversible, some mice were analysed more than 21 days after the insertion of the capsule. A histological examination of CRE activity, as assessed by expression of the LacZ [two mice (no. 1943 male and no. 1945 male with DOX, no. 1994 male and no. 1946 female without DOX; representative sections shown in Fig. 5)] or YFP gene (no. 1275 male and no. 1264 female with DOX, no. 1278 male and no. 1265 female without DOX; data not shown), was performed. Enzymatic analysis of β-gal activity was also analysed (four mice, no. 8157 female, no. 3472 female, no. 3478 male, no. 3468 male, representative section shown in Fig. 4b,c). Colocalisation of GnRH and CRE recombinase activity was assessed from β-galactosidase or YFP protein was observed in 88% of GnRH neurones (628 of 712 neurones, 89 GnRH neurones average per mouse with a SEM of 6.85 and an average of 78.5 neurones average per mouse with a SEM of 7.24 exhibiting Cre recombinase activity). There were no regional differences noted between CRE expression in the GnRH-CRE mouse (Fig. 2) or GnRH-CRETeR mice, and the GnRH-CRETeR CRE recombinase activity (Figs 4b,c and 5), except for the lateral septal population of cells (Fig. 4b). This lateral septal population of neurones was identical to those described above in the GnRH-CRE/ROSA26 mouse lines (Fig. 3). In mice bearing the GnRH-CRETeR transgene and the ROSA26 reporter locus, and not treated with DOX, no CRE recombinase activity was observed (Fig. 5, top right). In addition, there was no significant difference in the number of GnRH neurones in the DOX treated GnRH-CRETeR mice (89 ± 6.85 neurones per mouse) compared to the untreated GnRH-CRETeR mice (94.75 ± 6.3 neurones per mouse).

## Discussion

The CRE/*loxP* system provides a valuable method to produce cell- and tissue-specific gene deletions in mice. CRE, a bacteriophage recombinase, can mediate a synaptic union of two loxP elements located on the same linear DNA complex (11, 12). Thus, it has been used to delete the region between properly oriented LoxP sites in a specific gene (13, 15, 16, 22). *In vivo* recombination between *loxP* sites occurs with high efficiency if CRE is expressed at a sufficiently high level. For this reason, a number of transgenic mice have been produced in which CRE expression has been placed under the control of a cell- or tissue-specific promoter and used to target gene knockouts to specific cell types (15–17, 34). Systems have also been developed that allow temporal regulation of CRE expression, either by treatment with tetracycline or tamoxifen (22, 35, 36). The present study describes the development of two transgenic mouse lines: the GnRH-CRE line that expresses the CRE recombinase under the control of the GnRH promoter; and the GnRH-CRETeR line which expresses CRE in GnRH neurones following induction by tetracycline.

The GnRH-CRE line was developed by producing mice containing a transgene consisting of the CRE bacteriophage recombinase gene regulated by a 3446-bp fragment of the mouse GnRH promoter (Fig. 1a). This promoter region has previously been reported to be sufficient to target expression to GnRH neurones in the brain (5). The GnRH-CRE mice express CRE in GnRH neurones in the hypothalamus as assessed by double-label histological analysis (Fig. 2) while exhibiting virtually undetectable levels of CRE in other cells within the basal hypothalamus or in other tissues examined by western blot analysis (Fig. 1b). In addition, when GnRH-CRE mice are mated with the ROSA-LacZ reporter mice, offspring containing both the CRE transgene and the reporter locus possessed β-galactosidase expression in almost 87% of GnRH neurones, indicating sufficient GnRH neuronal CRE activity to induce recombination within the ROSA locus (Fig. 3). β-galactosidase expression was observed in a population of neurones in the lateral septum of mice that do not appear to express GnRH (Fig. 3). These neurones were also labelled in doxycycline treated mice containing the GnRH-CRETeR transgene and the ROSA-LacZ reporter gene (Fig. 4b). This suggests that the GnRH promoter activity in these neurones is ongoing and not due solely to an early developmental expression of GnRH in this population of cells as has been previously reported (33). These neurones may express a GnRH splice variant that is seen *in vitro* (37) or express GnRH that is not translated, or have a very low level of GnRH protein expression that cannot be detected using available GnRH antibodies. Support for ongoing expression in other populations of neurones comes from mapping studies in human brains in which population of GnRH expressing neurones were identified in the septum and dorsal preoptic area (38). Other groups have reported this population of neurones in the lateral septum expressing GnRH promoter reporter transgenes (6, 8, 18, 20, 21, 33). The significance of this population to reproductive development and function has yet to be determined. Since nearly all GnRH neurones are expressing CRE, the GnRH-CRE mouse is suitable for the constitutive disruption of genes in GnRH neurones when crossed with mice containing floxed alleles of expressed genes.

Other studies have produced mouse lines expressing CRE in GnRH neurones (18–21). The GnRH-iCRE mice produced by Shimshek *et al.* (18, 19) were produced using the same 3446 bp fragment of the murine promoter used to produce the GnRH-CRE mouse, but fused to an improved CRE gene (iCRE). These mice target CRE to GnRH neurones, but examination of CRE activity in reporter mouse studies using the ROSA26 reporter mice showed widespread CRE activity in the lateral septum, and in various other non-GnRH expressing cells located in the lateral hypothalamus, zona incerta, anterior hypothalamus, bed nucleus of the stria terminalis, caudate putamen, paraventricular thalamic nuclei, central amygdaloid nuclei, nucleus accumbens, posterior thalamic nuclei, paraventricular nuclei and the reticular thalamus. The GnRH-CRE/ROSA26 (Fig. 3) and GnRH-CRETeR/ROSA26 mice (Figs 4 and 5) reported in the present study exhibited far more restricted CRE activity. Although unproven, this may be a function of the modifications made to the CRE gene by Shimshek *et al.* (18, 19) that produced a more robust CRE species that can exert recombinase activity even in cells which have otherwise undetectable GnRH promoter activity.

The LHRH::CRE transgenic mouse produced by Yoon *et al.* (21) was constructed by inserting a CRE recombinase-poly(A) cassette into the first exon of the GnRH gene in a 212-kb BAC genomic fragment. Almost 100% of CRE expressing neurones in these mice expressed GnRH although whether nonbrain tissues were examined was not documented in this manuscript. In crosses with the ROSA26YFP reporter mouse (27), these mice also exhibited CRE activity in the lateral septum and bed nucleus of the stria terminalis, as seen in the GnRH-iCRE mice. Whether there was more widespread CRE recombinase activity in more caudal regions of the brain, as was reported in the GnRH-iCRE mouse, was not addressed. Importantly, the additional flanking regions of the gene present in the BAC transgene used by Yoon *et al.* (21) did not further restrict CRE activity compared to the GnRH-CRE transgene used in the present study, suggesting that the elements required to restrict expression are located in the proximal 3446 bps of the promoter.

A third mouse was reported as the present study was being written (20). These mice are reported to express CRE in 97% of GnRH neurones and to exhibit GnRH neurone-specific CRE recombinase activity in 97% of GnRH neurones as assessed in crosses with the ROSA26 reporter mouse. Few additional details were provided about these mice, and it is not clear as to what extent CRE was expressed in other neurones in the brain or in other tissues in the body. In addition, no mention was made of whether there was the CRE recombinase activity observed in non-GnRH neurones as reported by Yoon *et al.* (21), Shimshek *et al.* (18, 19) and ourselves.

The GnRH-CRETeR transgene was produced by subcloning the 3446-bp mouse GnRH promoter into the CRETeR vector (24). The proximal region of the promoter is modified to insert two Tet operators, binding sites for the Tet repressor (TetR). In addition, the transgene contains the TetR gene under the regulation of the CMV promoter, ensuring that the TetR protein will always be present to constitutively repress CRE expression. In the presence of tetracycline, or the more stable analogue doxycycline, the repressor is removed from the Tet operators, allowing for GnRH promoter driven expression of the transgene. We produced mice containing this transgene (Fig. 4a), the GnRH-CRETeR mouse, and have identified a line that expresses functional CRE protein, as assessed in the ROSA26 reporter mouse, in 88% of GnRH neurones in mice treated with doxycycline (Figs 4b,c). In mice not treated with doxycycline, CRE recombinase activity was not observed in GnRH neurones (Fig. 5). When induced, the degree of CRE activity is strikingly similar to the expression of CRE in the GnRH-CRE mice, indicating that neither the modifications to the proximal region of the GnRH promoter or differences in transgene insertion site have interfered appreciably with its function.

In summary, we report two mouse lines that express the CRE recombinase exclusively in GnRH neurones. The GnRH-CRE line expresses CRE solely under the control of the murine GnRH promoter. The second, GnRH-CRETeR, line is an inducible line that expresses CRE in GnRH neurones only following treatment of the mice with doxycycline. By crossing GnRH-CRE or GnRH-CRETeR mice with mice containing a floxed gene, the effects of the loss of the gene in GnRH neurones on the reproductive physiology of the animal can be studied. The GnRH-CRETeR mice would have the added benefit of temporally controlling the GnRH neurone-specific expression of CRE. Therefore, analysis of the role of a specific gene in GnRH neurones can be explored in adult mice without possible confounding developmental effects due to the absence of the gene. Furthermore, mice would be able to serve as their own controls by examining physiology before and after CRE recombinase induction. The availability of these mice will allow investigators to explore, *in vivo*, the importance of a vast array of genes on the central regulation of reproductive function.
